# The Italian food environment may confer protection from hyper-palatable foods: evidence and comparison with the United States

**DOI:** 10.3389/fnut.2024.1364695

**Published:** 2024-04-17

**Authors:** Tera L. Fazzino, Carmine Summo, Antonella Pasqualone

**Affiliations:** ^1^Department of Psychology, University of Kansas, Lawrence, KS, United States; ^2^Cofrin Logan Center for Addiction Research and Treatment, University of Kansas, Lawrence, KS, United States; ^3^Department of Soil, Plant and Food Sciences, University of Bari Aldo Moro, Bari, Italy

**Keywords:** food environment, carbohydrate, fat, sodium, sugar, fiber

## Abstract

**Background:**

Multi-national food corporations may saturate country-level food systems with hyper-palatable foods. However, the degree to which global food corporations have been integrated into country-level food systems may vary. Italy has largely retained local food production and may have low hyper-palatable food (HPF) availability in the food supply. The study quantified the prevalence of HPF in the Italian food system and compared the hyper-palatability of similar foods across Italy and the United States, which has wide HPF saturation.

**Methods:**

A national food system dataset was used to characterize HPF availability in Italy. A representative sample of foods commonly consumed in both Italy and the US were collected and compared. Foods represented six categories: cookies/biscotti, cakes/merendine, salty snacks, industrial bread, frozen pizza and protein/cereal bars. A standardized definition from Fazzino et al. identified HPF.

**Results:**

Less than one third (28.8%) of foods in the Italian food system were hyper-palatable. US HPF items had significantly higher fat, sugar, and/or sodium across most food categories (*p* values = 0.001 to 0.0001). Italian HPF items had higher fiber and/or protein relative to US HPF from the same category (*p* values = 0.01 to 0.0001).

**Conclusion:**

The Italian food system may confer protection from HPF exposure. HPF products in Italy had lower palatability-related nutrients and higher satiety-promoting nutrients.

## 1 Introduction

The industrialization of food systems globally has yielded substantial changes in country-level food environments and population health indices related to obesity and metabolic disease (1). However, there is variability in the degree to which globally produced foods have been integrated in different country-level food systems. Some countries, such as the United States (US), have developed a highly industrialized food environment run by several multi-national food companies (2) that have saturated the market with hyper-palatable foods (3). The food environment, combined with a structural environment that promotes limited physical activity and a culture that embraces convenience and eating on-the-go (4, 5) has yielded a US adult obesity rate 42.2%, the highest globally (6). However, other countries, such as Italy, have retained more local/national food companies and agricultural production and have experienced slower integration of industrialized foods into the food supply. Italy also has a structural environment that promotes physical activity and values food quality over convenience (7, 8). As such, Italy has one of the lowest adult obesity rates in the European Union (10.4%) (9, 10) and may represent an environment that confers greater protection from obesogenic foods.

To inform global obesity prevention efforts, it is important to identify food environments that are highly saturated with hyper-palatable foods, as well as those that confer protection from such foods. Hyper-palatable foods (HPF) contain combinations of palatability-inducing nutrients (fat, sugar, sodium, and/or carbohydrates) at thresholds that do not occur in nature, which yield a highly rewarding eating experience (11). HPF can excessively activate brain reward neurocircuitry, the same neurocircuitry activated by psychoactive drugs, and slow engagement of physiological satiety mechanisms (11–13). As a result, HPF may be difficult to stop eating and when consumed repeatedly over time, may increase the risk for weight gain and obesity. Prior research has identified the US food environment as being highly saturated with HPF. As of 2018, HPF comprised 68.9% of available foods in the US food system (3). Given the extensive HPF availability and the high rate of obesity among the US adult population (6), the US food environment may be considered obesogenic and therefore may substantially increase obesity risk.

In contrast, the Italian food system may represent a food environment that yields protection from HPF; however the availability of HPF in the Italian food system has not been quantified. Although not immune to the influence of the global food industry, the Italian food system has largely retained local and national-level food production, including food producers that specialize in key foods including breads, cheeses, and fruits and vegetables (7, 8). The structure of the food environment may facilitate the provision of high quality, fresh foods that are relatively inexpensive, consistent with an Italian cultural tradition that values fresh high quality foods and ingredients and that relies in a limited way on ready-to-eat, industrial foods (7, 8). Furthermore, southern regions of Italy have been recognized globally for their cultural dietary roots in the Mediterranean diet (14, 15), which is comprised of whole grains, legumes, and fruits and vegetables, combined with locally available fish, and limited intake of richer cheeses and cured meats (16, 17). Thus, the Italian food system may have a higher prevalence of whole, fresh foods that are not HPF (and correspondingly a lower prevalence of HPF). Additionally, evidence has indicated that some foods (e.g., fast foods) sold in different countries by the same parent company may have substantially different nutrient contents, and may be tailored to the country’s expectations surrounding taste preferences and health (18). Thus, HPF available in Italy may differ in their nutrient contents from the HPF available in the US, a premise that should be tested.

The purpose of the current study was to (1) quantify the prevalence of HPF in the Italian food system using nationally representative data obtained from the Banca Dati di Composizione Degli Alimenti per Studi Epidemiologici in Italia (BDA) (19); and (2) to compare the hyper-palatability of food products from categories that are commonly consumed in Italy and the US, using representative data collected from grocery stores in Italy and the US.

## 2 Materials and methods

The study was conducted in two parts and consisted of (1) analysis of an Italian food system dataset to quantify HPF availability nationally; and (2) data collection and analysis to compare the hyper-palatability of a representative sample of foods from Italian and US grocery stores. Procedures are detailed below. All data were processed and analyzed using R statistical software (20).

### 2.1 Processing and analysis of the national dataset

The study analyzed a dataset considered representative of the Italian food system, the Banca Dati di Composizione Degli Alimenti per Studi Epidemiologici in Italia (BDA) (19), to quantify HPF availability. The BDA was developed by a collaborative working group of researchers with the purpose of creating a database for use in epidemiological research (21). As such, the BDA is comprised of selected foods deemed to be representative of the Italian diet and the Italian food system on the national level (21). At the time of the study, the most recent update of the BDA was conducted in 2015 (19). The BDA provided detailed nutrient and ingredient data for a total of *N* = 978 food and beverage items. The BDA was processed in accordance with procedures from Fazzino et al (11) to apply the HPF definition to all foods. Beverages were removed before analysis, as the HPF definition does not apply to beverages (11). Thus, a total of *N* = 857 food items were analyzed. Total sugar was calculated by summing the values of glucose, fructose, galactose, sucrose, maltose, and lactose. Percent calories from fat, sugar, and carbohydrates (following the subtraction of fiber and sugar) were calculated. Salt was converted to sodium and then calculated as percent sodium per food weight in grams. Items that met criteria for at least one of the following were classified as HPF: (1) fat and sodium, FSOD (> 25% kcal from fat, ≥ 0.30% sodium), (2) fat and simple sugars, FS (> 20% kcal fat, > 20% kcal sugar), and (3) carbohydrate and sodium, CSOD (> 40% kcal carbohydrates, ≥ 0.20% sodium) (11). The percentage of HPF available in the BDA was calculated as n total HPF items divided by N total items. The percentage of each HPF group was also calculated using the same procedures.

### 2.2 Data collection and comparative analysis of Italian and US food samples

To directly compare similar food items available in Italy and the US, and to address a limitation of the BDA that it may not contain some prepared foods, we collected a representative sample of foods available in grocery stores from six categories of foods that were commonly consumed across both countries. Specifically, cookies/biscotti were considered dry sweet snacks that are commonly consumed in Italy (22–24) and the US (5, 25). Cakes/merendine were identified as moist sweet snacks and are commonly consumed at breakfast and as afternoon snacks across both countries (5, 26). Salty snacks included crackers, pretzels, breadsticks, and other crunchy savory items that are consumed as snacks or pre-meal appetizers across both countries (5, 24). Industrially produced breads were selected as a category because industrial breads are the standard bread product consumed in the US (4, 27), and while the consumption of artisanal breads is most common in Italy, the use of industrial breads is emerging (28). Similarly, frozen pizza was chosen as a category because it is widely consumed in the US (29) and its availability and consumption in Italy has grown in recent years (30). Finally, protein and cereal bars were selected because they have experienced wide expansion and consumption in both the US (31, 32) and Italian food markets (32, 33) and are marketed as a ‘healthier’ snack option than other available sweet or salty snacks (34). The foods across the six categories had similar methods of preparation, and were therefore directly comparable across the countries. Data were collected from grocery stores selected in the US and Italy, and data were collected on all products in the stores that aligned with the aforementioned six food categories. To collect food and nutrient data, researchers used mobile phones to photograph the front and back of all food items. Photographs were downloaded and food item and nutrient data were entered into excel spreadsheets using a standardized double-entry process.

Following data collection and entry, data were processed in preparation for applying the HPF definition. Percent calories from fat, sugar, and carbohydrates (following the subtraction of fiber and sugar) were calculated. Items that met criteria for at least one of the following were classified as HPF: (1) fat and sodium, FSOD (> 25% kcal from fat, ≥ 0.30% sodium), (2) fat and simple sugars, FS (> 20% kcal fat, > 20% kcal sugar), and (3) carbohydrate and sodium, CSOD (> 40% kcal carbohydrates, ≥ 0.20% sodium) (11).

A series of Fisher’s exact tests were used to compare the proportion of HPF items across Italian and US samples by food category. Fisher’s exact tests can be used to compare samples with different proportions and cell sizes (35), as was the case between Italian and US samples. In alignment with study aim 2 to examine differences in the hyper-palatability of items across Italian and US samples, we examined whether the Italian and US items had significantly different median values that contribute to HPF designation, specifically % kcal from fat, sugar, and carbohydrates, and % sodium per food weight in grams. Additionally, protein and fiber in grams were examined to understand potential differences in satiety-promoting nutrients across food categories and countries. The variables had different distributions and therefore Mood’s test of medians was used to compare Italian and US sample values for each nutrient of interest.

## 3 Results

### 3.1 National analysis

Findings indicated that 28.8% (247/857) of food items in the BDA met criteria as HPF, suggesting that less than one third of foods available in the Italian food system are hyper-palatable. The most common type of HPF was fat and sodium HPF (61.5%; 152/247). About a quarter of HPF items were fat and sugar HPF (24.5%; 61/247) and less than a quarter were carbohydrate and sodium HPF (20.6%; 51/247). Foods that were fat and sodium HPF were primarily preserved meats (e.g., cured pork) and cheeses (68.4%; 104/152). Items that were fat and sugar HPF were most commonly cookies and cakes (54.1%; 33/61) and items that were most commonly carbohydrate and sodium HPF were industrially produced breads and crackers (78.4%; 40/51).

### 3.2 Comparative analysis of Italian and US food products

Cookies/biscotti and cakes/merendine from Italy had a significantly lower percentage of items that were HPF relative to cookies/biscotti and cakes/merendine from the US (*p* values <0.001; Table 1). There were no other significant differences in the proportion of HPF for salty snacks, frozen pizza, industrial breads, and protein and cereal bars (*p* values = 0.081 to 0.999; Table 1) across countries.

**Table 1:** Prevalence of hyper-palatable foods among Italian and US samples.

	Italian	US	
	% HPF (n/N)	% HPF (n/N)	*p* value^a^
Food categories			
Cookies/biscotti	52% (14/27)	96% (196/205)	<0.00001
Cakes/merendine	77% (23/30)	100% (57/57)	0.0003
Salty Snacks	98% (54/55)	93% (654/700)	0.244
Industrial breads	94% (15/16)	95% (186/195)	0.554
Frozen pizza	100% (37/37)	98% (121/124)	0.999
Cereal and protein bars	68% (15/22)	84% (121/144)	0.081

a *p* value from fisher’s exact test.

HPF, hyper-palatable food.

Table 2 presents the food categories across HPF groups by country. Patterns across food categories were distinct; some food categories aligned primarily with one HPF group, others aligned with multiple HPF groups, and some patterns differed by country (Table 2). Across both countries, industrial breads were most commonly classified as carbohydrate and sodium HPF (Table 2). Cookies/biscotti and salty snacks from both countries were commonly classified as two HPF groups (Table 2). Furthermore, cakes/merendine from Italy were most commonly classified as fat and sugar HPF, whereas cakes/merendine from the US were commonly classified as both fat and sodium HPF and fat and sugar HPF (Table 2).

**Table 2:** Prevalence of hyper-palatable food groups among Italian and US samples.

	Italian			US		
	%(n/N)			%(n/N)		
Food type	FSOD	FS	CSOD	FSOD	FS	CSOD
Cookies/biscotti	54% (7/13)	62% (8/13)	23% (3/13)	63% (123/196)	84% (164/196)	9% (18/196)
Cakes/merendine	4% (1/23)	83% (19/23)	17% (4/23)	72% (41/57)	96% (55/57)	0% (0/57)
Salty snacks	43% (23/54)	0% (0/54)	98% (53/54)	83% (546/654)	5% (32/654)	61% (400/654)
Industrial breads	27% (4/15)	0% (0/15)	100% (15/15)	9% (16/186)	0% (0/186)	94% (175/186)
Frozen pizza	95% (35/37)	0% (0/37)	43% (16/37)	99% (120/121)	0% (0/121)	17% (21/121)
Cereal and protein bars	7% (1/15)	87% (13/15)	33% (5/15)	42% (51/121)	79% (95/121)	12% (15/121)

FSOD, fat and sodium hyper-palatable food group; FS, fat and sugar hyper-palatable food group; CSOD, carbohydrate and sodium hyper-palatable food group.

When examining HPF items specifically, US HPF items had significantly higher median values for at least one palatability-related nutrient, with the exception of industrial breads (Table 3). US HPF items had significantly higher % kcal (calories) from fat (salty snacks and frozen pizza) and/or % sodium (cakes/merendine, frozen pizza, cereal/protein bars; Table 3). US cookies/biscotti that were HPF also had significantly higher % kcal from sugar than Italian cookies that were HPF (35.8% vs. 25.3%; Table 3). Italian HPF items among cookies, salty snacks, and frozen pizza had significantly higher % kcal from carbohydrates compared to US HPF (Table 3). Italian industrial breads that were HPF had significantly higher % kcal from fat than did US industrial breads that were HPF (16.4% vs. 12.0%; Table 3). Regarding satiety-promoting nutrients, Italian cookies/biscotti and cakes/merendine that were HPF had significantly higher fiber than US items (Table 3), and Italian cookies/biscotti, cakes/merendine, and salty snacks that were HPF had significantly higher protein than US HPF items (Table 3).

**Table 3:** Comparison of hyper-palatable food items across Italian and US food samples.

	Italian	US	
	Median (IQR)	Median (IQR)	*p* value^a^
**Cookies/biscotti**			
% kcal fat	34.7 (6.5)	38.6 (11.3)	0.107
% kcal sugar	20.3 (5.7)	28.6 (11.4)	0.014
% kcal carbohydrates	31.0 (12.6)	26.3 (9.3)	0.006
% sodium	0.28 (0.15)	0.32 (0.12)	0.450
Total protein (g/100 g)	7.3 (1.8)	3.9 (3.3)	0.0003
Total fiber (g/100 g)	3.5 (1.8)	1.8 (3.3)	<0.0001
**Cakes/merendine**			
% kcal fat	38.7 (11.3)	41.3 (13.1)	0.323
% kcal sugar	25.3 (14.3)	35.8 (17.1)	0.138
% kcal carbohydrates	23.9 (12.5)	20.0 (7.8)	0.048
% sodium	0.20 (0.09)	0.35 (0.12)	<0.0001
Total protein (g/100 g)	7.2 (3.2)	3.6 (1.8)	<0.0001
Total fiber (g/100 g)	2.6 (1.8)	1.0 (2.1)	0.002
**Salty snacks**			
% kcal fat	24.5 (20.9)	45.0 (20.3)	<0.0001
% kcal sugar	2.9 (3.0)	1.4 (5.3)	<0.0001
% kcal carbohydrates	55.8 (13.5)	44.3 (15.9)	<0.0001
% sodium	0.70 (0.31)	0.70 (0.39)	0.440
Total protein (g/100 g)	10.1 (3.0)	7.1 (3.3)	<0.0001
Total fiber (g/100 g)	3.5 (4.7)	3.6 (4.1)	0.021
**Industrial breads**			
% kcal fat	16.4 (12.5)	12.0 (7.2)	0.001
% kcal sugar	4.9 (6.8)	10.0 (5.8)	0.059
% kcal carbohydrates	54.1 (12.5)	60.0 (10.3)	0.537
% sodium	0.55 (0.11)	0.47 (0.12)	0.073
Total protein (g/100 g)	7.7 (1.3)	9.5 (2.3)	0.009
Total fiber (g/100 g)	3.4 (2.8)	2.7 (4.8)	0.578
**Frozen pizza**			
% kcal fat	33.4 (8.6)	41.7 (8.0)	<0.0001
% kcal sugar	5.3 (2.8)	5.2 (3.4)	0.860
% kcal carbohydrates	39.3 (9.0)	33.1 (8.2)	0.0004
% sodium	0.47 (0.08)	0.52 (0.10)	<0.0001
Total protein (g/100 g)	10.0 (0.8)	10.1 (2.9)	0.014
Total fiber (g/100 g)	2.0 (0.8)	1.5 (0.3)	0.001
**Cereal and protein bars**			
% kcal fat	26.6 (10.7)	30.0 (14.3)	0.714
% kcal sugar	26.4 (15.2)	28.0 (9.0)	0.538
% kcal carbohydrates	20.6 (20.1)	28.0 (21.7)	0.584
% sodium	0.17 (0.18)	0.33 (0.15)	0.001
Total protein (g/100 g)	6.7 (6.9)	9.1 (14.7)	0.627
Total fiber (g/100 g)	4.3 (2.2)	4.2 (4.6)	0.809

a *p* value from Mood’s median test.

## 4 Discussion

The study examined the availability of hyper-palatable foods in the Italian food system and conducted the first comparative analysis of HPF across two countries, Italy and the United States. Findings revealed that less than one third of foods in the Italian food system were HPF, indicating that the Italian food system may confer some degree of protection from HPF exposure. Of the foods that were HPF, the majority were classified as HPF with elevated fat and sodium, and were typically cured meats and cheeses. A comparison of HPF among six categories of commonly consumed foods indicated that Italian cookies/biscotti and cakes/merendine had significantly lower proportions of items that were HPF, relative to US cookies/biscotti and cakes/merendine. Our findings also identified differences in the nutrient contents of HPF products across countries, with US products typically containing higher fat, sodium, and/or sugar, and Italian products typically containing higher carbohydrates and more fiber and protein. Taken together, findings indicated that HPF comprise less than one third of the Italian food system, and that HPF items from Italy tended to have lower palatability-inducing nutrients and higher satiety-promoting nutrients relative to US products that were classified as HPF.

Among the 28.8% of foods that were classified as HPF using the Italian national data, HPF items most commonly contained elevated fat and sodium, and were typically in cured meat products and cheeses. Most of the meat items that were classified as HPF had elevated sodium, which may have been necessitated by food safety considerations in the preparation process. Most HPF meats were prepared in a manner that involved slow aging of meat (e.g., curing) without direct cooking. To prevent the growth of bacteria or pathogens, sodium levels between 3.0 and 5.0% are typically required in cured meat products (36). Notably, the sodium level is in excess of the fat and sodium HPF criterion (≥0.30% sodium) and therefore it may not be surprising that many cured meats were classified as HPF. Overall, the finding that fat and sodium HPF was the most common type of HPF is consistent with prior studies conducted in the US food system (3, 11). Studies of the US food system also reported that meats and cheeses were commonly fat and sodium HPF; however most of the US produced meats were cooked and did not require high sodium content for food safety purposes, which may represent a difference across countries. Overall, evidence from two countries indicates that fat and sodium HPF may be the most commonly available type of HPF, and highlights meats and cheeses as commonly fat and sodium HPF. However, more work is needed to support this premise across countries globally.

Our findings overall revealed that most foods in the Italian food system do not have nutrient combinations that exaggerate their palatability, indicating that the Italian food system may confer some protection from HPF exposure. The finding is in stark contrast to the prevalence of HPF in the US food system, which demonstrated that as of 2016 (the year most closely matched to BDA 2015), 62% of foods in the US food supply were HPF (11). Thus, Italy had less than half of the HPF availability for the same time frame relative to the US. This study therefore presents the first evidence of different HPF availability across country-level food systems. Overall, the relatively low prevalence of HPF in the Italian food system and the high availability of whole fresh foods may protect the population from regular exposure to and consumption of HPF. The availability of non-HPF whole foods is consistent with Southern Italy’s cultural dietary roots in the Mediterranean diet (14, 15), which largely comprises whole grains, legumes, and fruits and vegetables, combined with locally available fish (16, 17). The low availability of HPF and adoption of the Mediterranean diet may promote higher diet quality and lower obesity, metabolic disease, and related chronic disease risk among the Italian population, which has been observed in the literature (15, 37). Furthermore, other characteristics of Italian societal structure and culture, including a built environment that facilitates physical activity (e.g., centralized towns built for walking, strong public transportation system), cultural preferences for high quality (non-HPF) food (7, 8), and limited reliance on eating outside of the home (7, 8) may contribute to the lower chronic disease rates as well. Overall, findings of the current study revealed the limited prevalence of HFP in the Italian food environment, a factor that is consistent with Italian dietary values and practices (7, 8), and may confer protection from obesity and chronic disease risk (15, 37).

In addition to analyzing nationally representative data, we also collected representative data from grocery stores in Italy and the US to compare products from six food categories identified a prior that are typically consumed in both countries that have similar preparation. Overall, there were substantially lower percentages of HPF among Italian cookies/biscotti and cakes/merendine relative to US cookies/biscotti and cakes/merendine. However, there were no significant differences in the proportion of HPF across Italian and US salty snacks, industrial breads, frozen pizzas, and protein and cereal bars. Findings regarding the substantially lower percentage of HPF among Italian sweet snacks (cookies/biscotti and cakes/merendine) may reflect a recent focus in Italy on ways to formulate products consumed by children to reduce child obesity risk. In a recent report by the World Health Organization, the overweight and obesity prevalence of Italian children was identified as among the highest of countries in the European Union (10), which has been attributed to a decreased adherence to the Mediterranean diet and reductions in physical activity (38). Thus, Italian food companies have focused on formulating products with greater care to help prevent child obesity, and their efforts may be reflected in these findings. However, we did not find any significant differences in the percentage of HPF among Italian and US salty snacks, industrial breads, frozen pizzas, and protein and cereal bars, many of which may also be consumed by children and contribute to obesity among children in Italy and the US. Thus, our findings highlight areas for potential improvement in both the Italian and US food industries regarding product formulation to promote health and reduce availability of HPF.

Our findings also indicated that HPF are not created equally, as evidenced by substantial differences across Italian and US foods that were classified as HPF. HPF items from the US had significantly higher contents of at least one palatability-related nutrient (fat, sugar, and/or sodium) across five of the six food categories, relative to Italian HPF items. HPF from Italy had significantly higher carbohydrates among three categories (cookies/biscotti, salty snacks, and frozen pizza), relative to US HPF items. Our characterization of carbohydrates in this study was focused on starchy carbohydrates, and did not include sugar or fiber. Therefore, our findings indicate that Italian HPF items had higher starchy carbohydrates in three of the six product categories relative to US HPF items. This finding is overall consistent with the Italian diet, which typically includes high quantities of starchy carbohydrates, such as pasta and bread (26). Therefore, starchy carbohydrates may be more accepted in packaged products such as cookies/biscotti and salty snacks as well. Furthermore, in the US and other European countries, low carbohydrate diets have become popular and starchy carbohydrate reduction may be a focus for consumers (39, 40). However, in contrast, Italians perceive a low carbohydrate diet as very far from their traditional food habits (41), a point that may also contextualize the differences in starchy carbohydrates across Italian and US products. Finally, Italian HPF had significantly higher fiber and/or protein across most food categories relative to US HPF items. Thus, Italian HPF items tended to have more satiety-promoting nutrients relative to US HPF items. Overall, findings indicated that Italian HPF had lower palatability-inducing nutrients and higher satiety promoting nutrients, relative to US HPF items.

The study had several limitations. First, the most recent nationally available data representing the Italian food system was from 2015. Therefore, it is unclear whether estimates of HPF availability may be different for today’s food environment. In addition, the BDA may have limited representation of prepared foods, which may lead to an underestimation of HPF availability. However, to address this limitation in the national data, we collected representative data from foods available in grocery stores in Italy and the US to directly compare foods from categories that may be underrepresented in the BDA, and that are commonly consumed across both cultures. Furthermore, to maintain methodological rigor in comparing foods across countries, we limited our comparisons to food categories for which the preparation was the same and for which the nutrient values would not change when cooked (e.g., frozen pizza).

In conclusion, our results indicate that HPF comprise less than one third of the Italian food system, indicating the Italian food system may confer protection from HPF exposure. Findings also revealed key differences in HPF products from Italy vs. the US, with HPF from Italy tending to have lower palatability-inducing nutrients and higher satiety promoting nutrients relative to US products of the same type. However, our findings suggest that food companies in Italy and the US should consider reducing the sodium, refined carbohydrates, and fat in salty snacks, frozen pizzas, industrial breads, and protein/cereal bars to reduce the hyper-palatability of these commonly consumed foods in Italy and the US.

## Data availability statement

The raw data supporting the conclusions of this article will be made available by the authors, without undue reservation.

## Author contributions

TLF: Conceptualization, Methodology, Investigation, Formal analysis, Writing – original draft. CS: Methodology, Investigation, Writing – review & editing. AP: Conceptualization, Methodology, Investigation, Writing – review & editing.
